# A Retrospective Study Using Mentzer Index for Prevalence of Iron Deficiency Anemia among Infants Visiting Maternal Centers at the Age of One Year

**DOI:** 10.1155/2022/7236317

**Published:** 2022-03-27

**Authors:** Johnny Amer

**Affiliations:** Department of Allied and Applied Medical Sciences, Division of Anatomy Biochemistry and Genetics, An-Najah National University, P.O. Box 7, Nablus, State of Palestine

## Abstract

Anemia, defined as a hemoglobin level two standard deviations below the mean for age, is prevalent in infants and children worldwide. Characterizing anemia as microcytic and normocytic depends on the mean corpuscular volume (MCV), which is an important parameter in differentiating many types of anemia. Microcytic anemia due to iron deficiency is the most common type of anemia in children. In this study, we aimed to assess the Mentzer index used by the Ministry of Health (MOH) in Palestine as a useful tool in differentiating between iron deficiency anemia (IDA) and thalassemia. We assessed for the prevalence of IDA among infants at the age of one year visiting the mother centers from seven West Bank provinces in Palestine. Medical records and hematology laboratory data of 3262 infants were retrospectively analyzed from the years of 2018 to 2020. The Mentzer index applied to all population by dividing mean corpuscular volume (MCV, in fL) by the red blood cell count (RBC, in millions per microliter). A corrected Mentzer index was further calculated among anemic infants to include only microcytic (MCV with less than 72 fl) and hypochromic (mean corpuscular hemoglobin concentration (MCHC) with less than 32 g/L) indices. Mentzer index calculations for the whole population showed that 29.1% were anemic (hemoglobin (HGB) less than 11 g/dl): 21.1% had mild anemia, 7.6% had moderate anemia, while 0.2% had severe anemia. The corrected Mentzer index calculations showed a prevalence of 5.9% and 3.2% among IDA and thalassemia infants, respectively. Severity of anemia was correlated with low body weight and infants born through cesarean mother birth with no interference with gender influence. CBC indices of RBC count, HGB, MCV, and mean corpuscular hemoglobin (MCH) showed a significant difference (*p* values < 0.05) between IDA and thalassemia infants' populations following the corrected Mentzer index. With the corrected Mentzer index, we introduced a new CBC index among infants at the age of 1 year in Palestine. These lab references could aid in differentiating IDA and thalassemia among the population and improve initial diagnosis screenings. The Mentzer index calculation for the whole population did not necessarily include cases of IDA, and therefore, it is recommended to comprise microcytic and hypochromic anemia indices prior to performing the Mentzer index.

## 1. Introduction

Worldwide, anemia is a major public health problem and affects up to one-half of children younger than five years [1–5]. Anemia in childhood is defined as a hemoglobin (HGB) concentration below cutoff levels established by the World Health Organization (WHO) less than 11 g/dl in children aged 6–59 months [6]. Microcytic iron deficiency anemia (IDA) is a common cause of childhood anemia, whereas macrocytic anemia is rare in children. IDA in infants between 6 and 12 months can be caused by getting less than the recommended daily amounts of iron. The recommended daily amounts of iron will depend on the child age and sex. From birth to six months, the recommended daily amounts of iron uptake in milligram (mg) is 0.27, and this amount is increased to 11 [7]. A diet that does not have enough iron is the most common cause. During periods of rapid growth, even more iron is needed [8]. Iron deficiency can be grouped into three categories according to severity: (1) biochemical iron deficiency with normal erythropoiesis, (2) biochemical iron deficiency plus iron-limited erythropoiesis but without anemia, and (3) biochemical iron deficiency with IDA9. A low serum ferritin and a low serum iron can identify biochemical iron deficiency. Iron-limited erythropoiesis can be recognized by a fall in both reticulocyte HGB content and mean corpuscular volume, without a fall in HGB or hematocrit (HCT) [9, 10].

Most infants and children with mild anemia do not exhibit overt clinical signs and symptoms. Initial evaluation should include a thorough history, such as questions to determine prematurity, low birth weight, diet, chronic diseases, family history of anemia, and ethnic background [11, 12]. A complete blood count is the most common initial diagnostic test used to evaluate for anemia, and it allows differentiating microcytic, normocytic, and macrocytic anemia based on the mean corpuscular volume [13]. In the current study, we assessed for the prevalence of IDA among children visiting mother care centers at one year of age using the Mentzer index.

## 2. Patients and Methods

### 2.1. Sampling

In this study, medical records of 3262 infants, aged 12 months, were obtained from seven mother centers in the West Bank (Palestine) provinces of (Nablus, Qalqilya, Tulkarem, Jenin, Ramallah, Bethlehem, and Hebron). Data were screened for IDA between the years of 2018 and 2020. 466 cases were randomly obtained retrospectively from each province. The data were collected through a systematic sample by each year and included RBC count, HGB, HCT, MCV, body weight, and type of delivery. The ethical committee of institutional review board (IRB) provided approval. Data obtained from each patient were summarized at the centers and were handled discretely. Names of the patients were kept anonymous.

### 2.2. The Mentzer Index

The Mentzer index was used in differentiating IDA from beta thalassemia [14]. The index depends on two parameters included from the complete blood count. If the quotient of the mean corpuscular volume (MCV, in fL) divided by the red blood cell count (RBC, in millions per microliter) is shown to be less than 13, it is likely that the patient could be thalassemic, while if the result is greater than 13, then the patient is most probably with IDA.

### 2.3. Mean Corpuscular Hemoglobin (MCH) and Mean Corpuscular Hemoglobin Concentration (MCHC) Calculations

MCH and MCHC were calculated from existing data for additional evaluation of IDA as hypochromic anemia: MCH = (hematocrit) HCT (%) × 10/RBC count (10–12/L) and MCHC = Hb (g/dL) × 100/HCT (g/dL).

### 2.4. Statistical Analysis

Data are shown as means ± SEM, unless stated otherwise. Statistical differences were analyzed between the IDA and thalassemia populations of the CBC indices using either the 2-tailed unpaired Student *t*-test (for comparison between two groups) or one-way analysis of variance (one-way ANOVA with Newman-Keuls posttests among multiple groups) using GraphPad Prism 5.0 (GraphPad software, La Jolla, CA). The *t*-test of *p* value below 0.05 was considered significant.

## 3. Results

### 3.1. Sample Characterization

**Figure 1:**
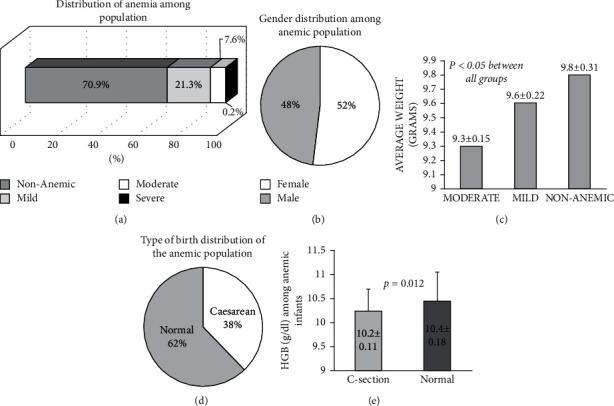
Demographic data of the infants: (a) Distribution of anemia among population according to WHO classification. (b) Gender distribution among anemic population. (c) Average weight of infants according to anemia severities. (d) Type of birth distribution of the anemic population. (e) Averages of HGB among anemic infants showing statistically significant results.

Blood testing for infants agedone year is a mandatory procedure performed at the Ministry of Health (MOH). In the current study, we aimed to assess the overall prevalence of anemia in our sample population. Infants' data were stratified according to HGB levels; HGB levels under 11 g/dl were considered anemia (10 to 10.9 g/dl as mild anemia, 7 to 9.9 g/dl as moderate anemia, and less than 7 g/dl reflected severe anemia). Infants with HGB levels above 11 g/dl were nonanemic.  Figure 1(a) displays that 29.1% of the infants were anemic (HGB less than 11 g/dl): 21.1% had mild anemia, 7.6% had moderate anemia, while 0.2% had severe anemia.  Figure 1(b) displays the gender of the population among anemic infants, showing that 52% were females while 48% were males. Next, correlation of anemia severities with the total body weight ± SD of the infants was performed.  Figure 1(c) shows inverse correlation between body weight and anemia severity among infants; significant results of *p* < 0.05 were obtained between the groups. Furthermore, attempt to relate type of birth recorded in infants' files with anemia was studied. Many studies demonstrated an association between cesarean delivery and anemia in infants and children [15]. In this study, the association of type of birth (normal vs. cesarean (C-section)) with the prevalence of anemia was assessed.  Figure 1(d) shows distribution of mothers' type of birth. Normal birth type was 77% and showed to be dominating the distribution among the nonanemic population (data not shown). Moreover, normal birth type distributions were reduced in the anemic population to 62% in favor of the C-section types. Average of HGB level in the C-section type was 10.2 ± 0.11 g/dl as compared to 10.4 ± 0.18 g/dl in the normal birth types (Figure 1(e), *p*=0.012).Additional CBC indices were included for better assessing anemia among infants' population.  Table 1 shows infants' population grouped according to normal and low levels of MCV, MCH, and MCHC. Of the population, 38.6%, 48.6%, and 18.2% had low MCV, MCH, and MCHC, respectively.

### 3.2. Prevalence of IDA and Thalassemia by Mentzer Index

In an attempt to calculate IDA prevalence in our all-infants population, the Mentzer index was applied; the index was used in mother centers of the MOH of Palestine, as indicated in methods. Surprisingly, around 90% of the total population exhibit a Mentzer index of >13 while 10% showed <13, indicating the overall prevalence of IDA and thalassemia, respectively (Table 2). In the USA, recent surveys documented that the prevalence of IDA in children aged one to five years is estimated to be 1% to 2%14. Therefore, obtained data revealed a high prevalence of IDA among Palestinian infants' population. In order to better interpret these data, the Mentzer index was calculated in the anemic vs. nonanemic population. Table 2 displays the anemic population demonstrating 83.2% of the population with Mentzer index >13 while 16.8% had Mentzer index <13. Moreover, 93.1% of the nonanemic population showed a Mentzer index of >13 and 6.9% had Mentzer index <13 (Table 2).

The above results reveal a high prevalence of IDA and thalassemia cases among the whole population whether they are anemic or nonanemic. In addition, these data indicate that HGB above 11 g/dl does not necessarily exclude cases IDA or thalassemia as indicated by Mentzer index. Data showed a high IDA prevalence among Palestinian infants with the use of Mentzer index. Moreover, the Mentzer index used by MOH mother centers showed surprising data of 16.8% of thalassemia infants, of which infants could be either carrier or diseased. These results, altogether, could raise concerns on using the Mentzer index as a sole indicator for initial screening and emphasize the need to include additional CBC indices that might help improving screening outcome.

### 3.3. Prevalence of IDA and Thalassemia by Using “Corrected Mentzer Index” and a New Generated CBC Reference Range

The classical diagnosis of IDA and thalassemia should include CBC indices of MCV, MCH, and MCHC. Therefore, from the infants' records, we were able to calculate MCH [HCT (%) × 10/RBC count (10–12/L)] and MCHC [Hb (g/dL) × 100/HCT (%)]. IDA and thalassemia both are classified as microcytic hypochromic anemia [16] with HGB, MCV, MCH, and MCHC relatively low in favor of thalassemia. Therefore, we reapplied a “corrected Mentzer index” and included infants exhibiting “microcytic hypochromic anemia” using following cutoff indices: HGB less than 11 g/dl (normal values 11–14.1 g/dl), MCV less than 72 fl (normal values 72–84 fl), MCH less than 25 (normal values 25–29 pg), and MCHC less than 32 (32–36 g/dl).  Table 3 displays CBC indices of the microcytic and hypochromic infants' population following the “corrected Mentzer index.” Calculated results of the “corrected Mentzer index” of the prevalence of IDA and thalassemia are summarized in Table 2 showing 5.90% and 3.20 %, respectively. Later on, CBC indices of RBC count, HCT, MCV, MCH, and MCHC were assessed in both infants' populations.  Table 3 summarizes averages of CBC indices in infants at the age of 1 year with microcytic and hypochromic anemia according to the “corrected Mentzer index”. Following the use of “corrected Mentzer index,” newly generated data were obtained and this included, for the first time, a new CBC reference range differentiating IDA and thalassemia that could be a useful initial screening for assessing anemia among infants at age 1 year.

## 4. Discussion

Anemia is a global public health concern [1–5]. The American Academy of Pediatrics (AAP) and the World Health Organization (WHO) recommend universal screening for anemia at one year of age 14. The recommended daily amount of iron for infants from birth to 6 months is 0.27 mg, and these requirements increase to 11 mg from the age of 7 months to 1 year [7]. Infants between 6 and 12 months demonstrated increased risk for iron deficiency, especially if they are fed with breast milk or using milk formula that is not fortified with iron. The iron that full-term infants have stored in their bodies is used up in the first 4 to 6 months of life. Breastfed babies who do not get enough iron should be given iron drops prescribed by their doctor [6].

Most infants and children with mild anemia do not exhibit overt clinical signs and symptoms [17]; this could mask initial diagnosis, thereby, preventing anemia consequences if not treated. Prevalence of IDA among the infants' populations could be attributed to inadequate dietary iron intake, feeding problems and noncompliance to treatment with no-follow up program, all of which demand an effective assessments strategy for minimizing IDA cases [1, 18]. In Palestine, mother centers and 1-year-old infants are screened for IDA and thalassemia through the Mentzer index. The Mentzer index was used in differentiating IDA from beta thalassemia [14]. The principle involved is as follows: in iron deficiency, the marrow cannot produce as many RBCs and they are small (microcytic), so both the RBC count and the MCV will be low, and as a result, the index will be greater than 13. Conversely, in thalassemia, which is a disorder of globin synthesis, the number of RBCs produced is normal, but the cells are smaller and more fragile [6, 19]. Hence, the RBC count is normal, but the MCV is low, so the index will be less than 13. Therefore, by using this index, the study aimed to evaluate the prevalence of IDA among children visiting mother care centers at one year of age in 7 major centers all across Palestine (West Bank and excluding Gaza strip). In these centers, gender of infants was recorded together with the type of birth of mothers (natural or C-section). Initial evaluation of family history, questions to determine prematurity, low birth weight, diet, chronic diseases, family history of anemia, and ethnic background was neither included nor registered. Concerning CBC indices, RBC, HGB, HCT, and MCV were obtained. Further calculations were made to calculate the MCH and MCHC, as indicated in the methods section.

Because of high prevalence of IDA and thalassemia among the anemic population of HGB levels above 11 g/dl, using the Mentzer index for the whole population could lack accuracy and might raise some concerns particularly in having false cases. Applying the Mentzer index among the nonanemic infants' population indicated existence of IDA and thalassemia cases that clearly reflects its noneffectiveness in assessing IDA and thalassemia among the general population. Therefore, attempts were made to reapply the Mentzer index on the infants' population exhibiting anemia of microcytic (low MCV) and hypochromic (low MCH and MCHC through manual calculations) indices (corrected Mentzer index). In Palestine, the prevalence of IDA is 5.9%, and this is 5-times higher than that in 1-year-old infants in the USA [13]. More than 40 mathematical indices have been proposed in the hematological literature for discriminating between IDA and thalassemia traits in subjects with microcytic RBCs. None of these discriminant indices is 100% sensitive and specific, and the ranking of the discriminant indices is not consistent [20]. The Mentzer index was used by mother centers of the MOH in Palestine, and the aim of this brief report was to better assess IDA between our populations.

## 5. Limitations and Conclusions

Although the current study showed no documented follow-up program for infants, where mothers of infants are asked to visit a family doctor for further evaluation and iron therapy, no evidence of visiting a family doctor or hematologist is known. Therefore, this report recommends a policy for follow-up to infants showing IDA and thalassemia through performing hemoglobin electrophoresis, blood film, and serum iron profile, including ferritin. Moreover, the study difficulty was gaining access to a list of a larger population, time, costs, and that bias can still occur under certain circumstances, especially that more data collection was influenced with COVID-19 epidemic restrictions. The study introduced new CBC indices among infants at the age of 1 year in Palestine that could be used as reference ranges to better identify/differentiate IDA and thalassemia among the population.

## Figures and Tables

**Table 1:** Infant population stratified according to CBC indices of MCV, MCH and MCHC.

Values (%)	MCV (72.7–86.5 fl)	MCH (24.1–29.4 pg)	MCHC (32.4–35.3 (g/dl)
Low	38.6	48.6	18.2
Normal	61.4	51.4	81.2

**Table 2:** Mentzer index in anemia and non-anemic populations. Mentzer index among all population, anemic population, non-anemic population and microcytic and hypochromic population.

Mentzer index	All population (%)	Anemic population (Hb < 11 g/dl)	N (%)on-anemic population (Hb > 11 g/dl)	Microcytic hypochromic population (MCV < 72.7 fl, MCH < 25 pg, MCHC < 32 g/dl)
>13	90	82.3	93.1	5.90
<13	10	16.8	6.9	3.20

**Table 3:** CBC indices among infants of IDA and thalassemia calculated by Mentzer index and applied on the classified microcytic and hypochromic anemia.

CBC indices	Thalassemia	IDA	*P* value
RBC count (10^6^/ml)	5.42 ± 0.46	4.9 ± 0.2	*P*=0.0001
HGB (g/dl)	9.5 ± 1.5	10.2 ± 0.7	*p*=0.03
HCT (%)	33.1 ± 3.3	33.7 ± 1.8	*P*=*ns*
MCV (fl)	61.1 ± 4.1	69.01 ± 2.2	*P*=0.02
MCH (pg)	18.1 ± 2.8	20.7 ± 1.4	*P*=0.04
MCHC (g/dl)	29.7 ± 2.2	30.2 ± 1.7	*P*=*ns*

## Data Availability

The descriptive statistics data used to support the findings of this study are included in the article. Data of the findings for this study are available from the corresponding author upon request.

## Abbreviations

IDA: Iron deficiency anemia
HGB: Hemoglobin
MCV: Mean corpuscular volume
MCHC: Mean corpuscular hemoglobin concentration
MCH: Mean corpuscular hemoglobin
WHO: World Health Organization
